# Speech sensorimotor relationships in francophone preschoolers and adults: Adaptation to real-time auditory feedback perturbations

**DOI:** 10.1371/journal.pone.0306246

**Published:** 2024-08-22

**Authors:** Paméla Trudeau-Fisette, Camille Vidou, Lucie Ménard

**Affiliations:** 1 Laboratoire de Phonétique, Université du Québec à Montréal, Montreal, Quebec, Canada; 2 Centre for Research on Brain, Language and Music, Montreal, Quebec, Canada; National Taiwan Normal University, TAIWAN

## Abstract

**Purpose:**

This study investigates the development of sensorimotor relationships by examining adaptation to real-time perturbations of auditory feedback.

**Method:**

Acoustic signals were recorded while preschoolers and adult speakers of Canadian French produced several utterances of the front rounded vowel /ø/ for which F2 was gradually shifted up to a maximum of 40%.

**Results:**

The findings indicate that, although preschool-aged children produced overall similar responses to the perturbed feedback, they displayed significantly more trial-to-trial variability than adults. Furthermore, whereas the magnitude of the adaptation in adults was positively correlated with the slope of the perceptual categorical function, the amount of adaptation in children was linked to the variability of their productions in the baseline condition. These patterns suggest that the immature motor control observed in children, which contributes to increased variability in their speech production, plays a role in shaping adaptive behavior, as it allows children to explore articulatory/acoustic spaces and learn sensorimotor relationships.

## 1. Background

Sensory feedback plays a crucial role in the development of speech mechanisms. During early life, the articulatory actions necessary for producing specific target sounds are linked to their corresponding sensory outcomes. These sounds can be both heard and felt, giving rise to auditory and somatosensory perceptual effects. In this way, developing children can store this auditory and proprioceptive information to construct a sensorimotor model of speech—a connection between articulatory movements and their acoustic and proprioceptive consequences [1–3]. The importance of auditory feedback in speech learning is demonstrated by the fact that children who are congenitally deaf and do not use hearing aids or cochlear implants fail to attain fully intelligible oral language [4–6].

In typically hearing children, acoustic feedback plays a crucial role in guiding their construction of a model to support speech fluency. Without adjustments in articulation, alterations of the shape, size, and strength of speech articulators could profoundly affect acoustic outputs [7, 8]. Once this model matures, the feedforward system takes over the control of articulators. Indeed, adult speakers no longer rely on the auditory feedback system, which is too slow for real-time use [1]. In neurotypical adults, the auditory feedback system’s role is primarily to update the model to ensure sensorimotor adaptation in challenging speaking conditions and to monitor speech so errors can be corrected [1, 8–10]. Despite the increasing body of research demonstrating that sensorimotor interactions exist early in life, the factors shaping those interactions remain largely unknown.

### 1.1 The real-time auditory feedback perturbation paradigm

In neurotypical adults, stored sensorimotor representations allow the brain to predict the effects of changes in the peripheral system (articulatory or sensory) and to correct for those changes. One set of experiments crafted to evaluate reliance on auditory feedback is the real-time auditory feedback perturbation paradigm. In this type of experiment, a speaker’s auditory feedback is modified with a digital processing card and sent back to the speaker through headphones. With current systems, the processing time is very short and the perturbation can be referred to as a “real-time perturbation.” Perturbations can be unpredictable, in that they occur randomly across trials, or they can be predictable, as they affect successive trials similarly [11, 12]. In the first case, the produced response is said to be reflexive and reflects the use of auditory feedback to adjust the production. In the latter case, responses are said to be adaptive and thus reflect speakers’ use of feedback-based correction and feedforward models [13–17]. An experimental phase, during which the perturbation is applied and maintained, informs about the participants’ use of auditory feedback to maintain and adjust internal models. When the perturbation is removed, the produced responses may differ from the baseline pre-perturbation and reflect long-term adaptation. In this paper, we focus on adaptive changes to predictable perturbations, as they provide a way to probe learning mechanisms. More generally, these response behaviors result from a multifaceted process that encompasses activities such as establishing sensory objectives, translating them into articulatory strategies, forecasting their sensory outcomes, perceiving sensory input, contrasting predictions with actual feedback, and engaging in inverse modeling to revise motor commands [18].

Whether the focus is on timing, volume, pitch, or formant frequencies, researchers have consistently shown that, when adult speakers are exposed to their own feedback with a real-time shift in a particular parameter, they mostly adapt their production by altering the affected parameter in the opposite direction to the perturbation (although they also occasionally mimic the perturbation, cf. [11]) [10, 19, 20]. Previous investigations have also revealed that the degree of response appears to be negatively correlated with the extent of the alteration: the larger the perturbation, the smaller the relative response [21, 22]. Furthermore, the responses are never entirely complete [21, 23, 24]. This adaptation typically becomes noticeable after several repetitions, when a certain level of perturbation is reached [21, 25, 26], indicating that the production-perception model tolerates some variability across trials, but adjustments to the production mechanisms become necessary beyond a certain level [17].

Furthermore, the magnitude of the response appears to be linked to the phonemic structure of the language, particularly in terms of vowel spacing. In languages where vowel systems exhibit varying degrees of proximity to neighboring vowels, such as French and Japanese, speakers exhibit different adaptive responses than English speakers [16, 27]. However, the effects of specific vowel distances seem limited within the same language, as shown by Nault and Munhall [28] and MacDonald et al. [21].

Other studies have shed light on the diverse individual factors that influence adaptation responses. First, researchers have delved into the impact of a speaker’s auditory perceptual abilities, including both acuity measures such as just noticeable differences (JND) and high-level perceptual abilities such as categorical perception. Concerning acuity, the underlying hypothesis is that individuals with superior perceptual acuity engage in more fine-grained auditory analysis, enabling them to detect minor disparities between altered feedback and expected feedback more effectively. This heightened ability tends to trigger more substantial sensorimotor responses than in speakers with lower perceptual acuity, who may struggle to perceive such discrepancies. One of the most frequently cited studies in this context is that of Villacorta et al. (2007) [10], which revealed a positive correlation between the extent of produced formant changes for perturbed F1 in the *head—had* continuum and the speaker’s JND in F1. Martin et al. (2018) [29] reported similar findings across three different measures of auditory perceptual acuity. On the other hand, Feng et al. (2011) [30] and Cai et al. (2012) [31] found no correlation between participants’ perceptual acuity and their response to auditory perturbations.

Another, somewhat related, factor that has occasionally been associated with sensorimotor adaptive behavior is within-speaker trial-to-trial variability for a given phoneme under normal conditions. For instance, Nault and Munhall (2020) [28] found that, although neither a speaker’s baseline variability (during a perturbation experiment) nor vowel spacing significantly predicted the degree of compensation along the F1 dimension in their multiple regression analysis, a significant Pearson correlation emerged between F1 within-speaker token-to-token variability and F1 changes. On the basis of a permutation test applied to the prior data, the authors however cautioned that this was most likely a chance result rather than a meaningful result, a conclusion in line with MacDonald et al. (2011) [24].

Lastly, sensory dominance, namely a speaker’s individual reliance on auditory versus somatosensory modalities, has also been explored in sensory perturbation experiments. In a study where both auditory and somatosensory perturbations were applied, speakers exhibited various adaptive responses in the two modalities: all speakers adapted to at least one of the two perturbations but some of them showed a preference for somatosensory adaptation [32]. This between-speaker variability in sensory preference suggests that speakers may possess specific weightings for each modality [33], with primary reliance on auditory feedback being more prevalent [30].

### 1.2. The case of speech development

Despite the critical role of auditory feedback in speech development, the exact factors shaping the children’s use of sensory feedback and feedforward models are not well understood. In a meta-review of perturbation studies on pediatric populations, Coughler et al. (2022) [12 report on 14 studies that involved real-time perturbation of one or two formant frequencies [34–48].

Among those studies, MacDonald et al. (2012) [34] reported that adults and preschool children (4 years old) exhibited similar opposing patterns in response to simultaneous F1/F2 manipulation of the English vowel /ɛ/. These results support the idea that auditory feedback plays a crucial role in monitoring and correcting speech production errors in adults and that this ability is well developed in preschoolers. However, toddlers (2 years old) did not adapt to the formant shifts, and substantial within-participant token-to-token variability was observed in the younger speakers. The authors noted that production variability, indexed by the standard deviation of produced F1 and F2 values in the baseline phase, decreased with age, implying an age-related aspect of the utilization of auditory feedback for speech production control. Similar results regarding the adult-like magnitude of adaptation responses were reported in subsequent studies of typically developing children ranging in age from 3 years-old to 11 years old [35–37, 44, 46, 48, comparing the typically developing groups]. A somewhat different pattern is reported in a study of Dutch children and adults (van Brenk and Terband (2020) [38]), in which it was found that although children (aged 4 to 9 years) and adults produced comparable F2 responses, larger F1 changes are found in children compared to adults. Moreover, unlike adults, children maintained their adaptation behaviors even when auditory feedback was restored to normal. Children also displayed greater token-to-token variability throughout the experiment, leading the authors to conclude that linguistic representations may be less stable in children than in adults. Larger token-to-token variability in the baseline phase was also reported in 5- to 12-year-old children than in adults [42], but there was no correlation between this variability and the magnitude of the adaptation. This pattern contrasts with that obtained by Coughler et al. (2021) [43], who found that such variability is related to a decrease in the magnitude of response in children (6 to 11 years old) (see also [49, 50] on F0 perturbation). Although no significant group differences were reported between 9- to 11-year-old children and adults in response to altered centroid frequencies of /s/, Shiller et al. (2010a) [44] showed that sensorimotor training had a more limited effect on children’s categorical perception boundary compared to adults. The authors interpreted this result as suggesting that auditory feedback might play a limited role in children’s perceptual representations. It is noteworthy that the amount of adaptation in preschoolers was related to their literacy skills in studies of French, English, and Dutch [37, 40, 41]. Moreover, even though behavioral responses to altered formant frequencies are comparable in children and adults, they rely on different cortical structures involving distinct patterns of connectivity [41, 42].

The paradigm involving predictable real-time auditory feedback perturbation has also been used to explore children’s sensorimotor representations in clinical speech [43, 46, 47]. Mixed results were reported: children with speech disorders produced larger adaptive responses or, on the contrary, smaller responses than their typically developing peers. The key point is here that responses to such feedback manipulations are an index of mature sensorimotor representations and control.

While additional studies are necessary to gain a more comprehensive understanding of the role of auditory feedback in speech motor control development, prior research indicates that young children are generally capable of adapting to alterations in their auditory feedback and feedforward commands similarly to adults. However, the significant variability in their productions and their early learning behaviors suggest that their auditory-motor connections have not yet fully matured. Furthermore, since the factors deemed to affect adaptive responses in adult speakers (e.g., variability, perceptual acuity, vowel spacing, sensory reliance, etc.) are also at play during speech development in children, similar response patterns exhibited by both adults and children may arise from the influence of different factors.

## 2. Objectives

The goal of this article is to further explore the development of sensorimotor interactions by comparing preschool-aged children’s and adults’ adaptation strategies in response to auditory-feedback manipulations of formant frequencies in French. This study is part of a larger project investigating the effects of multimodal sensory development on speech production in Quebec French. Complementing our previous studies ([51, 52]), we chose to focus our attention on the rounding contrast between the phonemes /ø/ and /e/, a phonological feature that has rarely been investigated in acoustic manipulation studies. The production of this contrast also provides auditory, somatosensory and visual cues that are likely used as targets or templates in the course of development.

First, we sought to investigate whether preschool-aged children and adults would adapt their productions to a similar degree when confronted with altered auditory feedback. In light of previous work, our hypothesis was that both children and adults would be able to alter their production as a response to the formant manipulation, but that children’s productions would be more variable ([53, 54]). To further explore the efficiency of the participants’ sensorimotor representations, the links between the magnitude of their adaptive responses and the following variables were tested: within-speaker variability, vowel spacing, and participants’ own perceptual abilities.

## 3. Methods

The research protocol was approved by Université du Québec à Montréal’s Institutional Review Board (no. 2016–700). Data collection took place between April 15, 2019, and December 1, 2019.

### 3.1. Participants

Sixty-five native speakers of Canadian French were recruited for this study. Six potential participants were excluded due to equipment malfunction (1 adult), inability to perform the task (2 children) or poor data quality (3 children). In the end, 30 adults (aged 19–30; 13 females; mean age = 26.2 years, SD = 4.0) and 29 preschoolers (aged 4–6; 15 females; mean age = 5.2 years, SD = 0.7) were included in the analyses.

All participants were tested for their pure-tone detection threshold (DT) using an adaptive method (DT < 25 dB HL at 250, 500, 1,000, 2,000, 4,000 and 8,000 Hz) and had normal or corrected vision. They (or their parents) reported having no speech, language, psychological or neurological disorders and gave written informed consent to the experiment.

### 3.2. Experimental procedures

The task presented in this study was administered in a three-experiment session in which adults and children were asked to perform two bimodal perceptual tasks (one audiovisual, one audio-somatosensory, counterbalanced order across participants) and one production task. This article discusses the results of the production task, in which real-time auditory perturbations were applied.

#### 3.2.1 Production task: Real-time auditory perturbation

First, a picture-naming task was administered. In this task, participants were required to name cartoon characters by their names, featuring the vowels /I a u e ø/. This task was used as a familiarization task to make sure the participants remembered the target names.

Then, participants had to produce 70 utterances of the rounded vowel *eu* /ø/, as in the word *eux* (“them”). The vowel corresponded to the name of a cartoon character used before in the familiarization task (although participants did not know that the name represented the vowel being tested). The task was to say the character’s name each time it appeared on the screen. That way, we made sure that they were producing the vowel in the appropriate timeframe, and it made the task more appealing for the children.

In Montreal French, the contrast between /e/ and /ø/, in acoustic space, is mainly realized through F2: lip rounding when going from /e/ to /ø/ typically decreases the length of the front cavity with which F2 is affiliated. Note that a slightly more back position of the tongue is observed in /ø/ than /e/, which likely contributes to lengthening the front cavity. Thus, throughout the experiment, the produced vowel /ø/ was gradually shifted toward /e/, by increasing each individual’s F2 by 40% using Audapter [55]. The decision to manipulate auditory feedback by percentage rather than through absolute formant values ensures that change better respects the nonlinear processing of frequency by the human ear. Because children aged 4 to 6 have higher spectral values than adults, a 100 Hz manipulation (for example) would have been proportionally greater for adults than for children. To constrain formant measurements and avoid errors, the mean individual F2 values produced for /ø/ in the familiarization experiment were calculated and included in the automatic formant detection algorithm.

**Fig 1:**
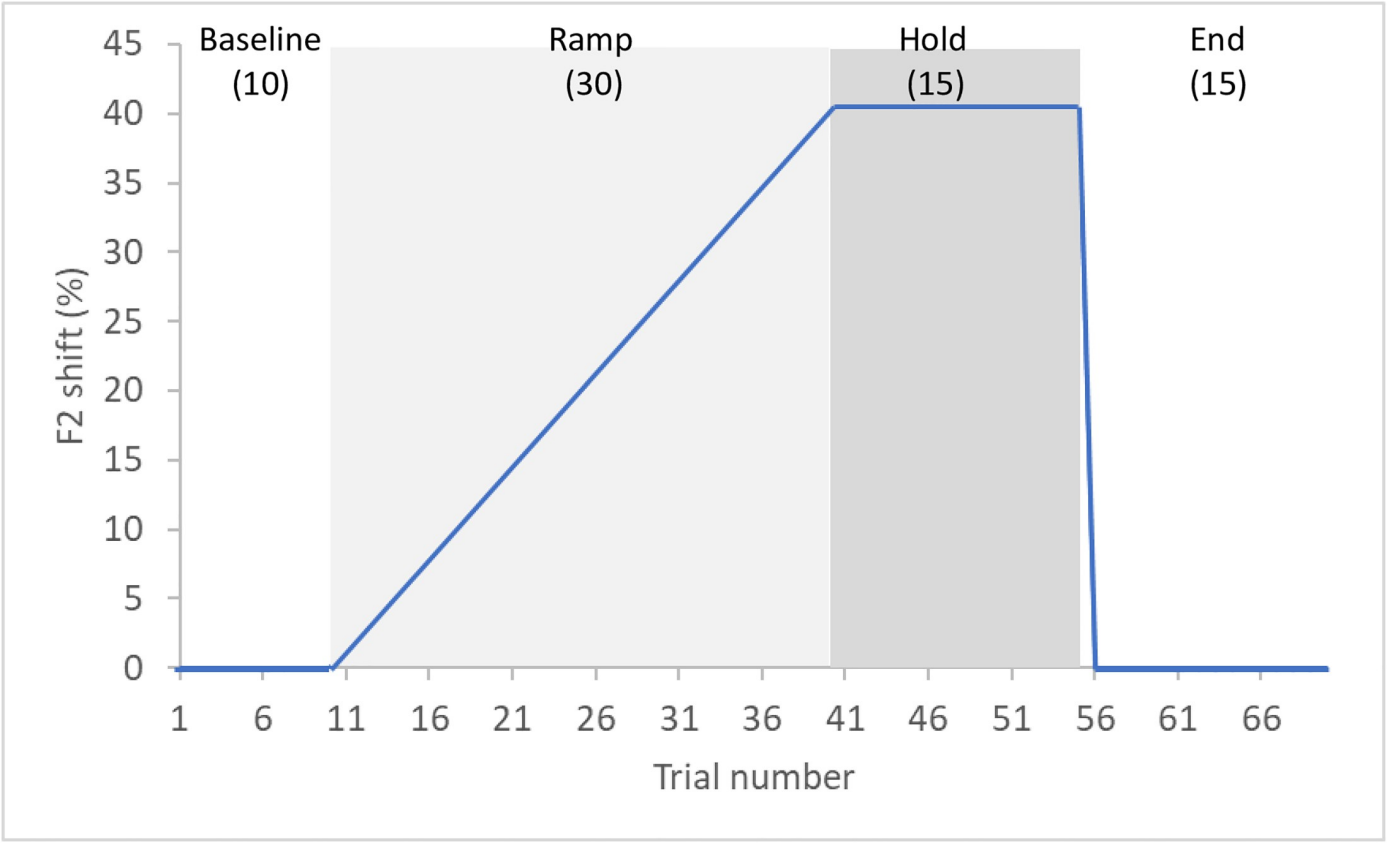
Schematic representation of the acoustic manipulation throughout the four experimental phases.

The experiment was divided into four phases (Fig 1). In the first phrase (Baseline: 10 utterances), participants produced the vowel *eu* /ø/ ten times and received normal auditory feedback. In the second phase (Ramp: 30 utterances), participants received altered auditory feedback during which F2 was incrementally increased by 1.33% in each trial (the other formants were not shifted). Therefore, in the 4^0t^h trial, participants received auditory feedback in which F2 was raised by 40%. To do so, we set the Audapter vector length (pertAmp parameter) at 0.4 and the angle (pertPhi) at π/2 radian. In the third phase (Hold: 15 utterances), the maximal 40% upward shift was maintained in all trials. In the 5^6t^h trial, the perturbation was abruptly removed. Thus, during the last phase (End: 15 utterances), participants received normal auditory feedback. Throughout the experiment, auditory feedback was amplified using a Roland Duo-Capture Ex amplifier and mixed with white noise (at a level of 40 dB SPL), to mask bone-conducted feedback. In the familiarization task, we made sure participants produced vowels loudly enough so that the resulting signal-to-noise ratio was approximately 30 dB SPL. Acoustic signals were recorded with a high-quality Audio-Technica microphone (Omnidirectional condenser headworn microphone, model number BP892) and digitized at 44100 Hz using a Delta 1010 LT sound card with a buffer size of 128 samples. The total feedback loop latency was 34 ms, in the range of values provided in [56].

#### 3.2.2 Perceptual task: Auditory identification

The auditory perceptual data used in this paper come from a subtask presented in Trudeau-Fisette et al. (2019) [51], in which the effects of somatosensory perturbation on auditory perception were investigated in the same participants. We used such a test instead of a just noticeable difference (JND) test to measure high-level auditory processing (in line with [44], which are still being finely-tuned in children. In the Baseline condition of the task reported in Trudeau-Fisette et al. (2019) [51], each participant was instructed to identify the vowel they perceived and asked to choose between /e/ (the vowel used in the name of a fairy, French *fée* /fe/) and /ø/ (the vowel used in the name of a fire, French *feu* /feu/). The stimuli were 10 synthesized vowels equally stepped in F2 between the two end-point stimuli /e/ and /ø/ using the Maeda model [57]. Each stimulus was presented 10 times, and all stimuli were presented in random order. Note that only a subset of participants took part in this perceptual task (16 adults and 14 children). Note that all tasks were presented to each participant in the same order, within a single visit: the perceptual tasks were thus always performed before the formant perturbation task (in line with [37]). This was done in order to avoid influencing the perceptual scores with the perturbation task, as demonstrated in previous research specifically targeting this effect ([44]). Furthermore, pilot experiments revealed that children’s attention was too difficult to maintain when the perceptual tasks were following the production task. All participants were encouraged to take breaks between tasks.

### 3.3 Data analysis

#### 3.3.1 Production data

Each of the 70 utterances of the vowel /ø/ was manually segmented with Praat software. The mean duration of vowels was 326 ms for adults and 385 ms for children. Using a customized script, the first four formants were first extracted at the vowel midpoint. Formant values were inspected and errors were corrected manually. The formant values and the auditory inputs and outputs of each of the child and adult participants were also carefully examined. For three children, it appeared that the acoustic manipulation was not performing properly (error with noise generation or stimuli presentation). Thus, those three participants were excluded from the analyses. For each individual, the mean F2 values in the 10 utterances of the Baseline trials were calculated. Those values were used as a baseline to calculate the ratio of formant values relative to the Baseline for each subsequent trial. For each phase, within-speaker trial-to-trial variability corresponded to the standard error of normalized F2 ratios during that phase. To compare values across individuals and phases, a linear model was built in R (using the *lm* function) with standard error values as the dependent variable and Group and Phase as the independent variables. For each of the four experimental phases, values corresponding to the first three trials and to the last three trials were averaged. It is true that the first and last trials do not refer to the same trial numbers in each phase (as is usually the case for developmental auditory feedback perturbation experiments) but they nonetheless allow an assessment of changes throughout the phase and comparison of those changes when feedback is shifted (Ramp phase, for instance) with changes when feedback is not perturbed (Baseline phase, for instance). To assess the impact of the auditory perturbation across experimental phases, F2 ratios collected from the 59 participants were fitted into a linear mixed effects model (LMEM; using the *lme4* package in R [58]) in which the fixed factors were Group (Adults or Children), experimental Phase (Baseline, Ramp, Hold, End), and Trial number (first three trials and last three trials) and the random factor was the individual participant. Post hoc analyses were performed using multiple comparisons (using the *multcomp* package in R).

#### 3.3.2 Perceptual data

The percentages of vowel stimuli perceived as /e/ in the auditory identification functions were analyzed using the Probit regression model, implemented in Matlab. For each participant, the slope of the labeling function and the 50% crossover category boundary were extracted, in line with previous work showing developmental changes and interindividual differences in both dimensions (boundary and slope; e.g., [59, 60]). Group differences were assessed for each of the dependent variables (category boundary and slope) using linear models in R (Jamovi), with Group as a fixed factor.

#### 3.3.3 Multiple linear regressions

Finally, to investigate to what extent variability, vowel spacing in the F2 dimension, and identification scores could predict the compensatory formant values produced, we ran a multiple linear regression (MLR) using the *lm* function in R. To compute speakers’ variability in the F2 dimension, we calculated the standard error values across the 10 trials in the Baseline. Vowels produced in the pre-experiment session (see previous section) were also analyzed to calculate vowel spacing between /e/ and /ø/. For each repetition, F2 values were extracted in Praat using the same algorithm mentioned before. After normalization using *z*-scores, the difference in F2 between /e/ and /ø/ was computed and used as an index of vowel spacing. For both children and adults, an MLR was performed to predict normalized F2 values in the Hold phase from the following predictors: trial-to-trial variability, vowel spacing in F2 between /e/ and /ø/, and perceptual identification performance (slope of the category boundary) between the vowels presented in Trudeau-Fisette et al.’s study [51] for 16 adults and 14 children.

## 4. Results

### 4.1. F2 values produced

**Fig 2:**
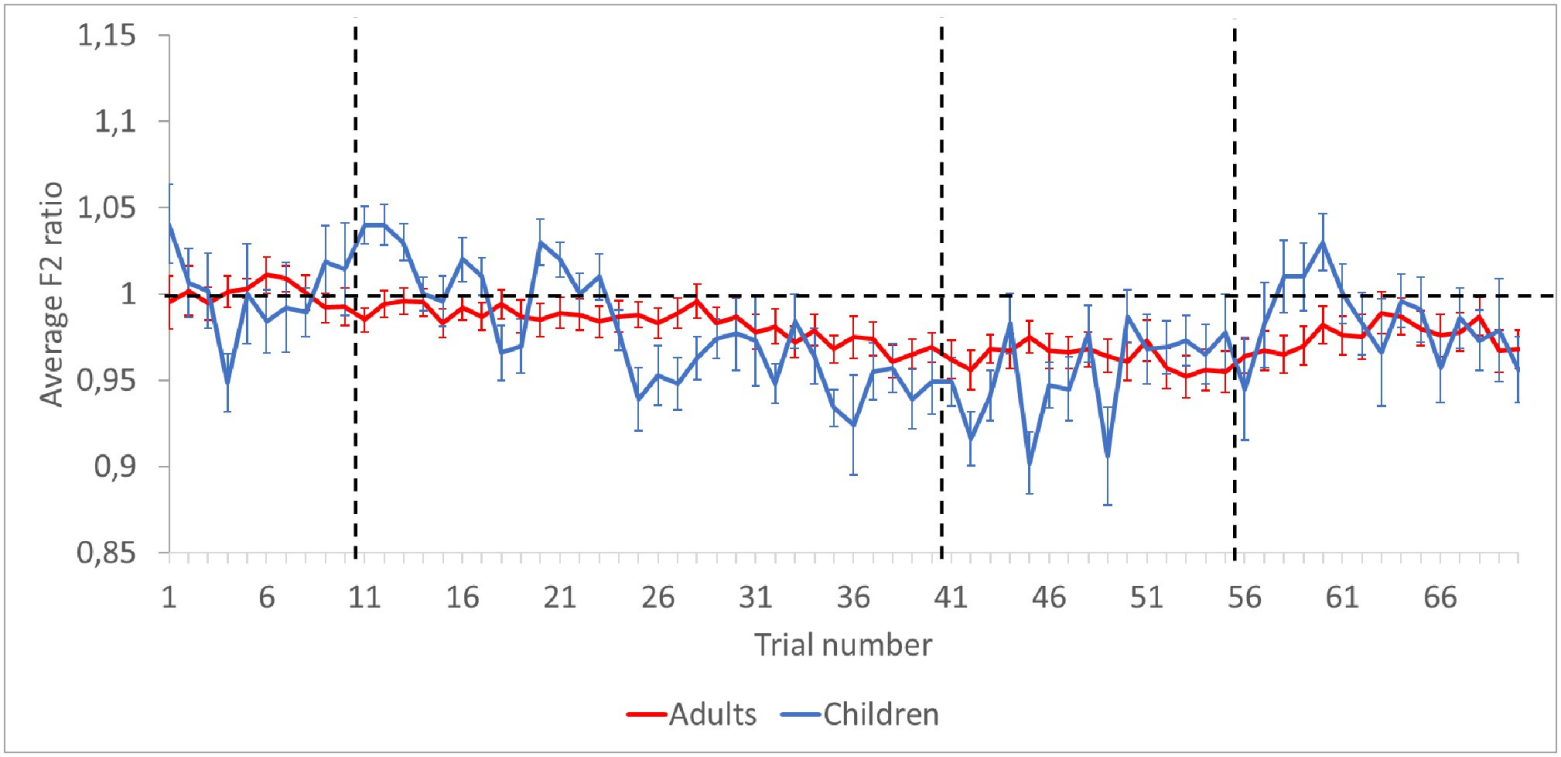
Mean F2 ratios for each trial. Data are averaged across speaker groups. Error bars indicate standard errors.

Fig 2 displays the normalized F2 values for each trial and for both speaker groups, averaged across speakers. Since the degree of adaptation is presented as a ratio, a value of 1 refers to productions for which there is no change compared to the baseline. Values greater than 1 indicate that there has been an increase in the observed formant (following response), while values less than 1 denote a decrease in this formant (opposing response).

Fig 2 shows that both speaker groups, on average, produce opposing responses to the auditory perturbation in the Ramp and Hold phases, as expected. The LMEM on F2 ratios revealed significant main effects of Phase (*F*(3,1323.2) = 14.23, *p* < .001) and Trial (*F*(1,1323.1 = 4.98, *p* < .05), a significant two-way interaction effect between Phase and Trial (*F*(3,1323) = 8.94; *p* < 0.001) and a significant three-way interaction of Group, Phase and Trial (*F*(3,1323) = 4.57, *p* < .01). Multiple comparisons were computed to further explore this three-way interaction and revealed that, while both child and adult groups gradually decreased their F2 from the beginning to the end of the Ramp phase in order to cancel out the perceived acoustic manipulation (adults: *t* = –3.57; *p* < 0.01; children: *t* = –5.77; *p* < 0.001), the children also significantly increased F2 in the first trials of the Ramp phase (*t* = 2.16; *p* < 0.05). This pattern suggests that, when encountering an auditory perturbation resulting in increased F2, children first exhibit a following response rather than an opposing response. By the end of the Ramp phase, both speaker groups had achieved the same amount of F2 changes. In the Hold phase, when the F2 perturbation was maintained at its maximum level, both speaker groups produced mean F2 values that were significantly lower than in the Baseline (adults: *t* = –3.60, *p* < 0.001; children: *t* = –5.32, *p* < 0.001). At the end of the Hold phase, children increased their F2 but did not return to baseline values (*t* = 2.64, *p* < 0.01). In the End phase, once the manipulation was turned off, children and adults maintained F2 values that were significantly lower than in the Baseline (adults: *t* = –2.52, *p* < 0.05; children: *t* = –3.40, *p* < 0.001). It is interesting to note, however that speakers showed a tendency to first produce F2 values quite similar to those of the Baseline, and then move away again from the Baseline values. We shall return to this point in the discussion.

**Fig 3:**
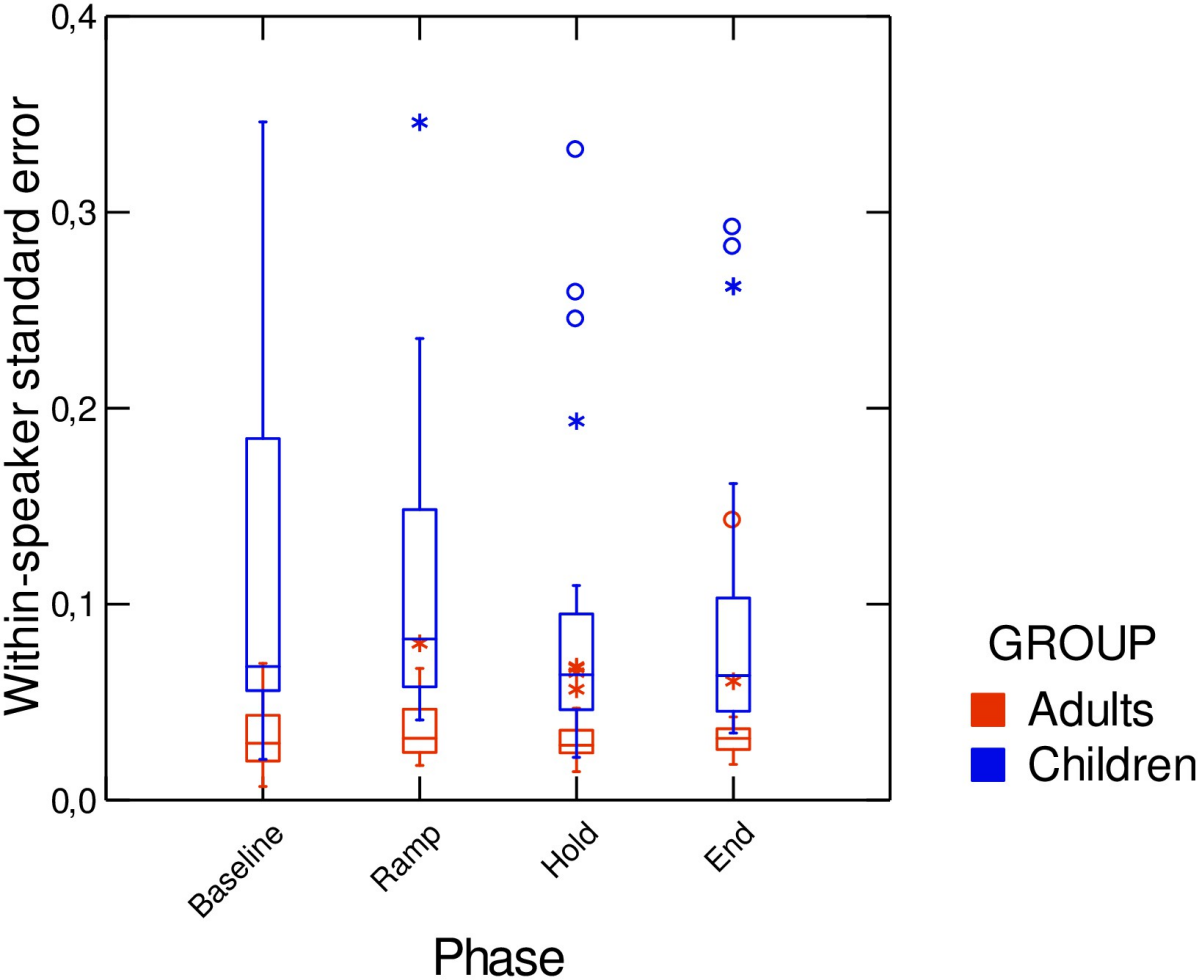
Mean values of standard error in F2 produced in the baseline, for both speaker groups. Error bars are standard errors.

Data from Fig 2 also suggest that variability is greater in children than in adults. In order to compare the extent of within-speaker variability across trials in the four experimental phases, standard errors across normalized F2 ratios within a given phase were computed. Data are displayed in Fig 3. Linear models confirmed that children exhibited significantly greater within-speaker variability than adults, across all phases (*F*(1,228) = 86.18; *p* < 0.01).

**Fig 4:**
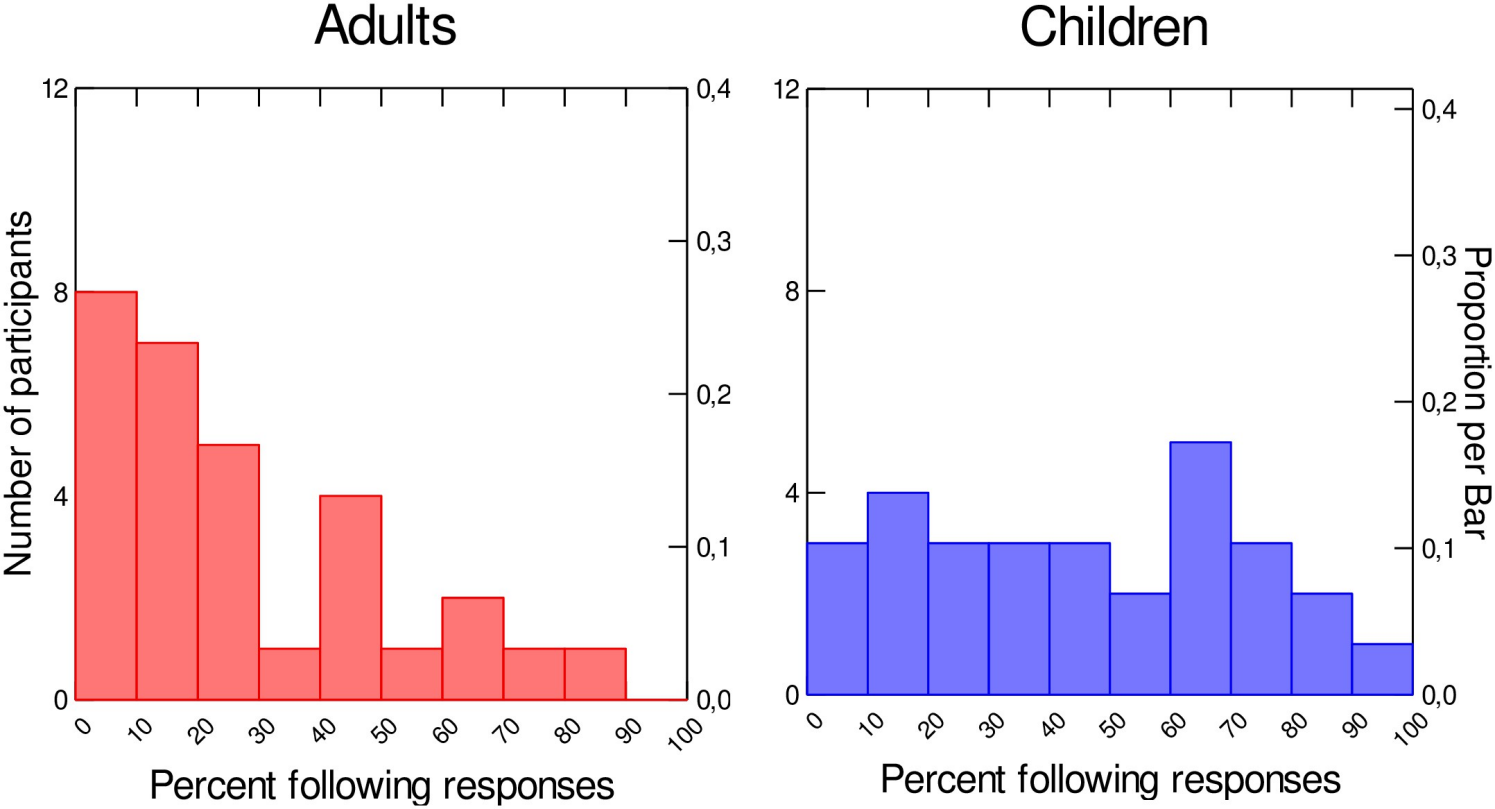
Distribution of speakers according to the percent following responses (F2 ratios>1) in the Ramp phase.

To further explore how speaker’’ responses to the auditory manipulation are distributed, for each participant the percentages of trials in the Ramp phase for which F2 ratios are greater than 1 (indicating a following response) were calculated. Fig 4 presents a histogram of all participants percent following responses, broken down by group. As shown in Fig 4, in the adult group (left panel), more than 66% of participants (20 out of 30) produce fewer than 30% following responses, while 34% of children (10 out of 29) enter that category. On the contrary, 45% of children (n = 13) produce more than 50% following responses in the Ramp phase, a much higher proportion than adults (n = 5, 17%). As in [24], children produced more following responses than adults.

### 4.2. Vowel spacing

**Fig 5:**
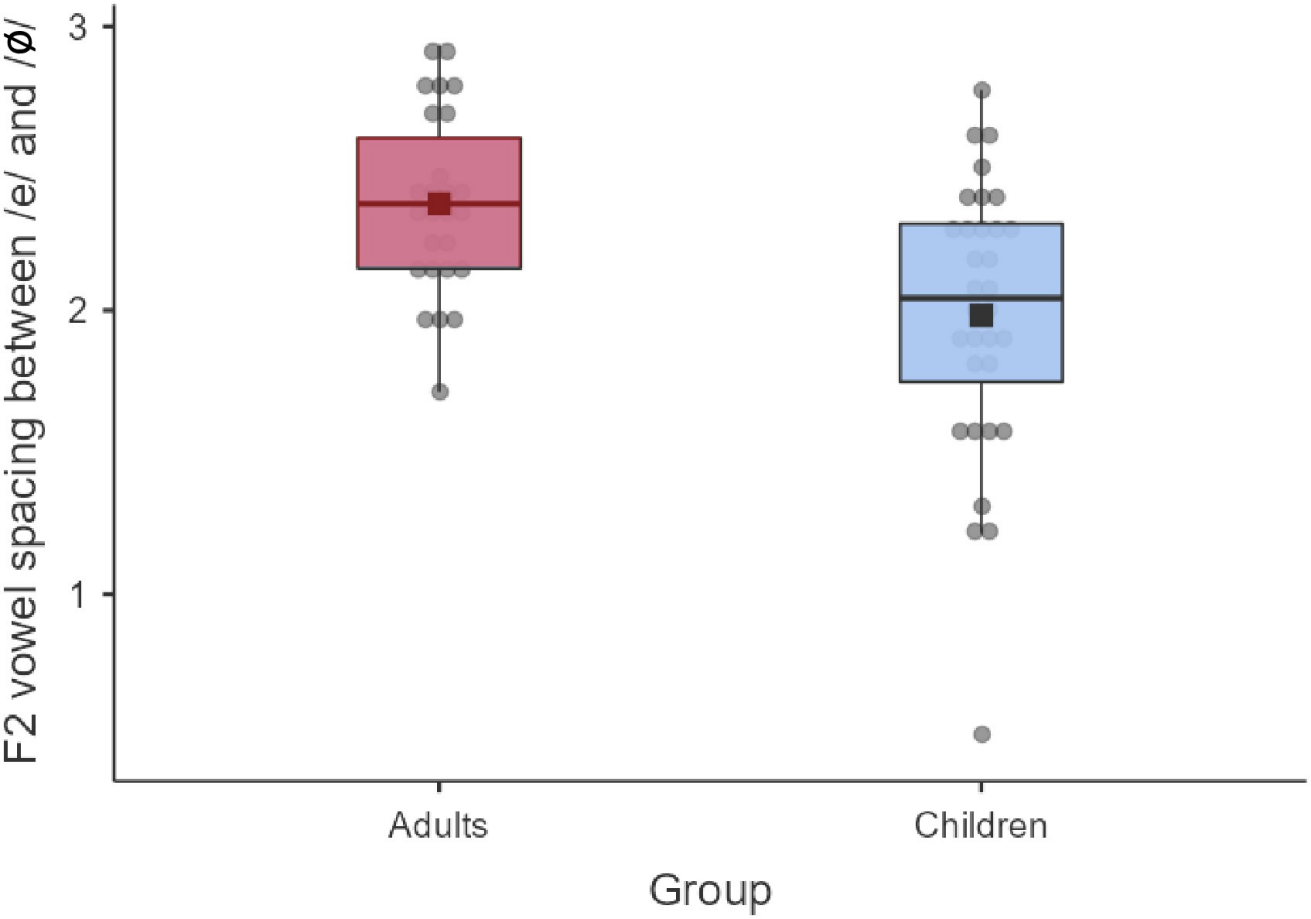
Mean difference between /e/ and /ø/, in *z*-scored values of F2, across speaker groups. Error bars are standard errors.

Apart from trial-to-trial variability in the Baseline, another variable that was used to describe speakers’ production space was vowel spacing between /e/ and /ø/ targets in the F2 dimension. Fig 5 presents the mean difference between the two vowels’ *z*-scored F2 values, across speaker groups. As suggested by the figure, adults produced larger F2 differences between /e/ and /ø/ than children (*t* = –2.97; *p* < 0.01).

### 4.3. Auditory perceptual data

**Fig 6:**
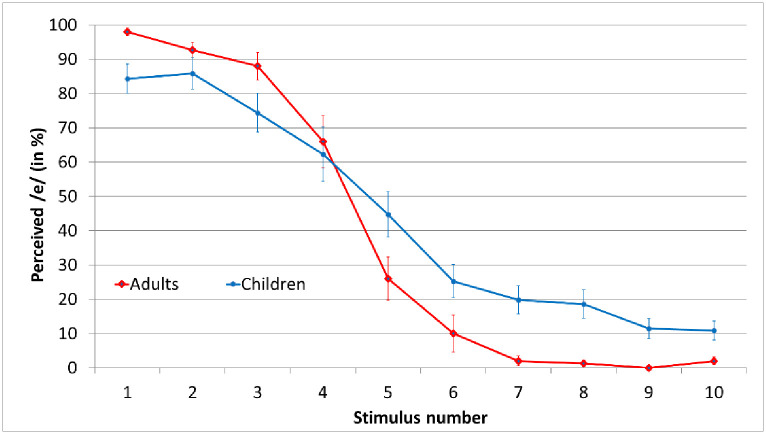
Average psychometric functions for both speaker groups.

**Fig 7:**
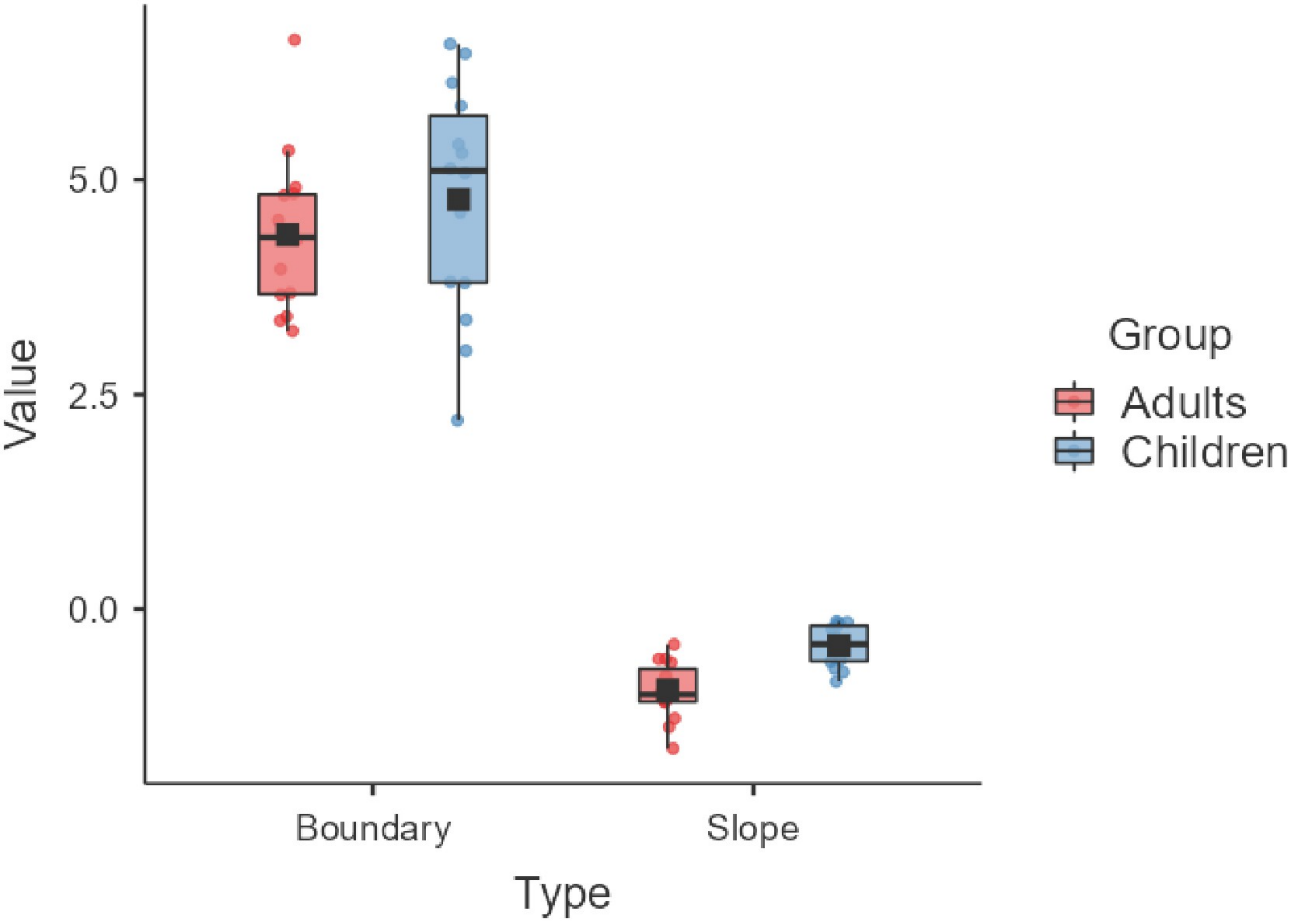
Slope and category boundary of the labeling function obtained for each participant, averaged across groups. Error bars are standard errors.

When we consider the auditory identification functions that were obtained from the participants prior to the production task (see Fig 6), different group patterns are observed. Values of slope and category boundaries are displayed in Fig 7. Regarding the slope of the labeling function, adults had significantly larger absolute values than children, suggesting that their slopes were steeper than the children’s (*t* = 4.80; *p* < 0.001). This is in line with previous research suggesting that children engage in less categorical perception than adults. Turning now to category boundary, no significant differences were found between the two speaker groups.

### 4.4. Predictors of compensatory responses

To identify which factors might predict the magnitude of participants’ adaptation responses, two multiple linear regression analyses (MLR) were conducted, one for each speaker group. The dependent variable was the mean value of F2 achieved in the Hold phase, and the predictors were the speaker’s trial-to-trial variability in the Baseline (indexed by standard error), the slope of the labeling function, and vowel spacing. Table 1 presents the standardized beta weights associated with each parameter. Recall that, because of the direction of the auditory perturbation, opposing responses correspond to values lower than 1; a negative correlation therefore means that lower F2 ratios (indicating stronger adaptation responses) are related to higher values of a given variable. For the adult speakers, the regression model was significant (*p* < 0.001) and accounted for 59% of the variance in F2 (a percentage higher than that reported in Nault and Munhall, (2020) [28]). As shown in Table 1, of the three factors included in the model, only the slope of the labeling function had a significant effect (*p* = 0.005): speakers who had steeper slopes produced lower F2 ratios. This factor predicted 53% of the variance. For the child group, the overall model was also significant (*p* < 0.001) and accounted for 57% of the F2 variance. In contrast with the adult data, only F2 variability in the Baseline phase had a significant effect on F2 (43% of the variance): speakers who displayed more variability also produced lower F2 ratios.

**Table 1:** Regression coefficients, *t*-values and *p*-values for the multiple regression analyses, in 16 adults and 14 children.

	**Estimates**	***t*-values**	***p*-values**
	**Adults**		
**Intercept**	0.89	7.99	< 0.001*
**Slope, labeling function**	–0.14	–3.53	0.005*
**F2 Baseline variability**	–1.06	–1.51	0.160
**Vowel spacing**	0.02	0.47	0.650
	**Children**		
**Intercept**	0.67	5.35	< 0.001*
**Slope, labeling function**	–0.38	–2.02	0.071
**F2 Baseline variability**	–3.72	–2.73	0.021*
**Vowel spacing**	–0.04	–0.34	0.740

## 5. Discussion

The aim of this study was to investigate the development of sensorimotor relationships by observing how strategies employed by preschool-aged children and adults differ when they must adapt to manipulations of formant frequencies in auditory feedback. Our primary objective was to determine whether both children and adults would exhibit similar levels of adaptation when faced with altered auditory feedback in a predictive paradigm. Building upon previous research, our first two hypotheses posited that both children and adults would be capable of adapting to the formant manipulation, though children’s productions might display greater variability. Subsequently, we aimed to ascertain whether both groups would apply similar strategies in adapting their productions and responding to the perceived altered feedback. Given that acoustic-motor connections may not have fully matured in preschool children, we hypothesized that the two experimental groups’ sensorimotor responses would be related to different variables.

### 5.1. The effect of the auditory feedback perturbation on F2 values

In this study, based on the F2 values of the produced vowels (see Fig 2), children and adults exhibited a comparable magnitude of adaptation to the perturbation. Indeed, by the end of the Ramp phase, when the acoustic perturbation was at its maximum, speakers from both groups had reached a similar relative F2 value. This finding aligns with previous studies, which have generally indicated that children and adults use similar adaptation strategies [34, 44, 46, 50]. This value was also maintained at the onset of the Hold phase, again at a similar level for all participants. Thus, despite immature motor control abilities and representations, children were able to adapt to the perturbation. In essence, they utilized their auditory feedback control subsystem to rectify their actions over the course of the perturbed trials and updated their feedforward command subsystems to predict the sensorimotor response, much as adults did. When the manipulation was turned off (in the End phase), according to the LMEM, all participants maintained lower F2 values, resembling what they had produced during the Hold phase. This pattern likely reflects the learning process that occurred during the perturbation, wherein internal sensorimotor models (feedforward commands) were updated based on feedback manipulation. As noted earlier, speakers showed a tendency to quickly return to the Baseline F2 values in the End phase, and then move away from them again. This pattern could be interpreted as reflecting the speakers’ broader sensorimotor representations. Indeed, the Ramp and Hold phases led to a remapping of articulatory gestures on their sensory consequences: novel lip and tongue positions were then incorporated into the representations. Once the feedback perturbation was removed, speakers still used those novel gestures for a few trials. Although this is a possible explanation for the variability of the production during the End phase, it does not explain why speakers showed a trend to first return to the Baseline target and then move away from it again. This is a pattern that may be better understood following future studies with a larger number of trials in the End phase.

Despite these similarities, certain differences were observed in the adaptation profiles. Initially, during the Ramp phase, the vowels produced by children displayed F2 values that mirrored the induced perturbation (increased F2), whereas the vowels produced by adults during the same timeframe were already countering the perturbation (decreased F2). In addition, a distribution chart of F2 ratios suggested that, throughout the Ramp phase, children produced more following responses than adults. Although it has been reported elsewhere that following responses are common in experiments with adults (see for instance [11], albeit to a much lesser extent than opposing responses), Liu et al. (2010) [61] also found that children displayed a larger proportion of following responses than adults in a pitch perturbation study. An increase in following responses has also been noted in some clinical populations (speech sound disorder, hyperfunctional voice disorder, etc.; [36, 62]). It is likely that this pattern reflects the immaturity of internal models during early speech development. This result is in line with a previous study of ours, which found that somatosensory information played a greater role in adults’ speech than in children’s, suggesting that the integration of auditory and somatosensory information (i.e., development of internal speech representations) evolves throughout the course of development ([51]).

### 5.2. Variability and learning

In line with our second hypothesis, our results also showed that young speakers were more variable than adults. Those observations support previous findings of greater variability in children’s productions [24, 38, 44, 50]. It is generally assumed that motor control development is associated with a significant reduction in trial-to-trial variability, in line with current models of motor control development [53, 54]. Thus, it is striking that children’s variability in our results was correlated with the mean amount of adaptation they produced in the Hold phase. This pattern of results might be ascribed to the important role variability likely plays in sensorimotor mapping. Indeed, on the one hand, the detrimental effect of immature motor control variability induces noise in motor commands and/or acoustic output; however, on the other hand, such variability is believed to facilitate motor exploration toward a specific goal and improve learning [63].

In their investigation of the role of short-term changes in speech motor performance related to practice, Walsh et al.(2006) [64] found that children demonstrated greater plasticity in speech-motor learning, allowing them to acquire and store audio-articulatory mappings more quickly than adults. Shiller et al. (2010a) [44] also reported that centroid manipulation led to a larger boundary shift for adults than for 9- to 11-year-olds, and that 5- to 7-year-old children benefited greatly from relevant perceptual training, leading to stronger responses to F1 perturbation. In our experiment, the fact that children who had greater within-speaker variability exhibited larger mean adaptation responses, which persisted even after the perturbation had been removed, suggests that their sensorimotor models were more affected by the previous productions [10, 16, 38] than the models of those who displayed less variability, and thus had less opportunity to map large sensorimotor spaces.

### 5.3. Factors shaping compensatory behaviors

In order to assess the role of perceptual abilities, vowel spacing and within-speaker variability in speakers’ responses, MLR analyses were performed. The results showed that the main predictor of the mean value of F2 changes in the Hold phase in adults was perceptual abilities (as indexed by the slope of the category function between /e/ and /ø/), whereas in children it was within-speaker variability (as indexed by the standard error values in F2 ratios in the Baseline). This finding for adults is in line with that of Nault and Munhall (2020) [28], who found a similar correlation among their adult participants in the F1 dimension, and with findings reported by Daliri and Dittman (2019) [65]. The fact that variability was a significant predictor of adaptation response for children suggests, on the contrary, that variability might be beneficial for some aspects of speech development, as suggested above.

### 5.4. Limitations of the study and future directions

One possible limitation of this study is that the vowels produced in the different phases of the experiment were not perceptually assessed. In a follow-up study, identification tests and quality rating tests could be used to determine whether the response patterns induced by the articulatory maneuvers were successful in producing the perceptual speech goal associated with /ø/. Another potential limitation is the small number of trials during the four phases of the experiment. Even though this design was chosen to make sure the task would not be too long for children, it could be improved by providing reinforcement during the experiment. Thus, more trials could be added. Also, in our experimental design, the perceptual task was always performed prior to the production task. Despite our best efforts to minimize the effects of one task on the following one by introducing long enough breaks between tasks, we cannot rule out the possibility that the measured effects were influenced by the previous tasks. This limitation, however, does not contradict our main results, since such an order effect would have been similar for all participants. Furthermore, this order ensured the perturbation task itself did not induce any shift in perceptual functions (cf [44]). More diverse measures of perceptual skills, such as just noticeable differences, could have been collected to assess lower-level acuity, which is necessary to detect auditory perturbation. Finally, articulatory data could also be collected using ultrasound imaging combined with Optotrak motion tracking, for instance, as a way to analyze the exact articulatory maneuvers involved in the production of the vowel under the different feedback conditions.

## 6. Conclusions

The results of this study indicate that preschool-aged children show overall similar adaptation but greater variability in speech productions than adults when dealing with altered auditory feedback. The substantial variability of children’s productions suggests that their internal models of speech are not as robust as those of adults. However, such variability is found to be related to the amount of adaptation in children, suggesting that this pattern is beneficial to sensorimotor mapping and learning. Adults’ magnitude of F2 adaptation, on the other hand, was correlated with the slope of their auditory categorical function.
